# Supplementary material for: In vivo proteomic mapping through GFP-directed proximity-dependent biotin labelling in zebrafish
Source: eLife. 2021 Feb 16;10:e64631. doi: 10.7554/eLife.64631 (PMC7906605; doi:10.7554/eLife.64631)

Figure 1 - source file 1

Figure 1B: Streptavidin-HRP blot

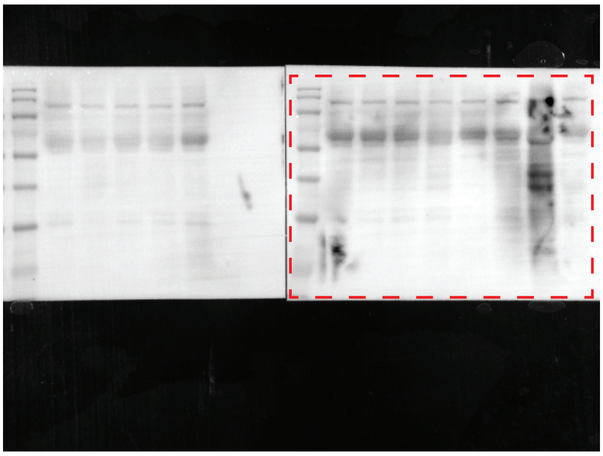

Figure 1B: Anti-Myc immunoblot

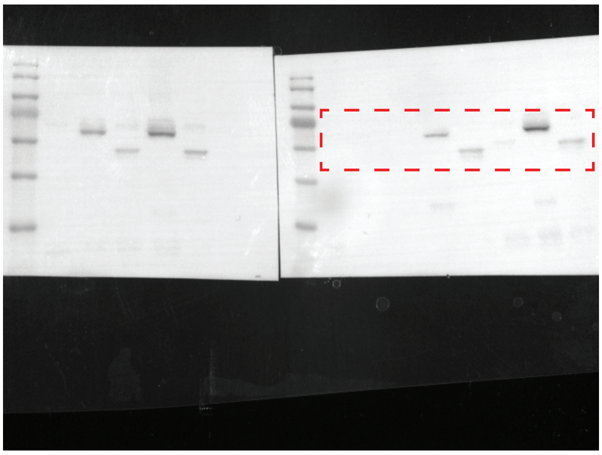

Figure 1B: Anti-Actin immunoblot

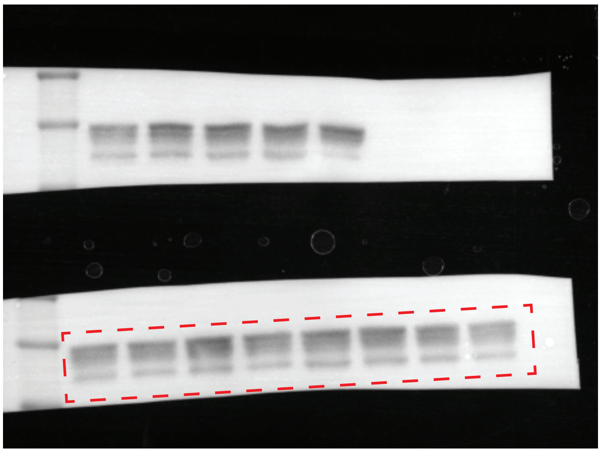

Figure 1D: Streptavidin-HRP blot

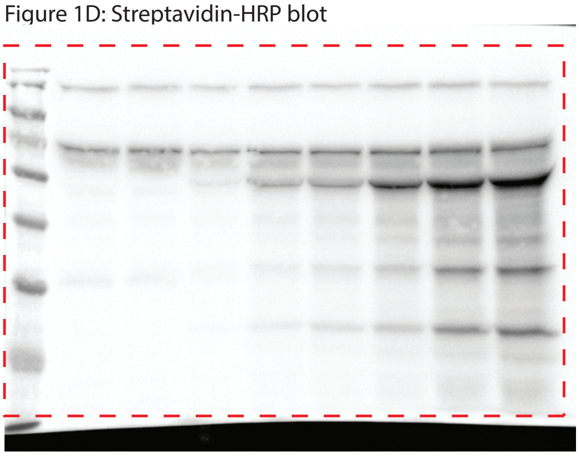

Figure 1D: Anti-Myc immunovblot

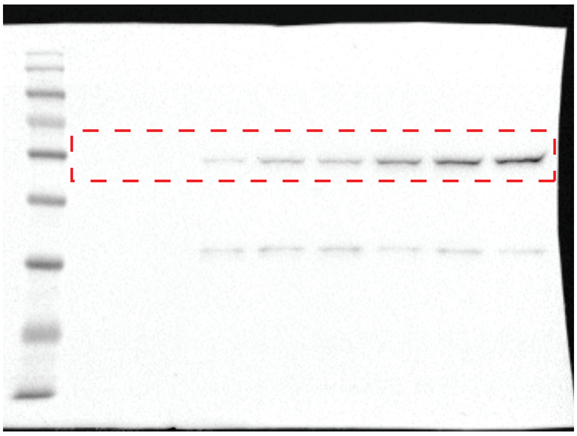

Figure 1D: Anti-Actin immunoblot

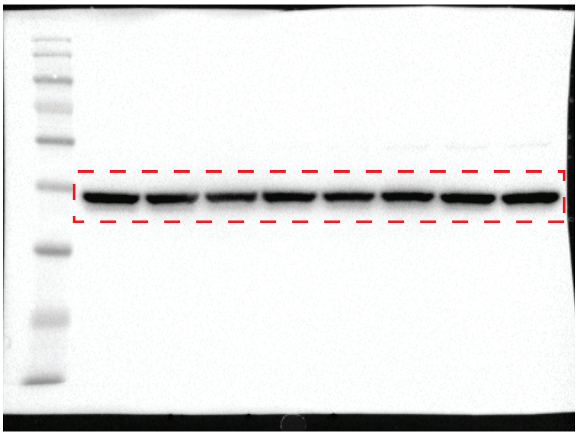

Figure 1 - source file 1

Figure 1B: Streptavidin-HRP blot

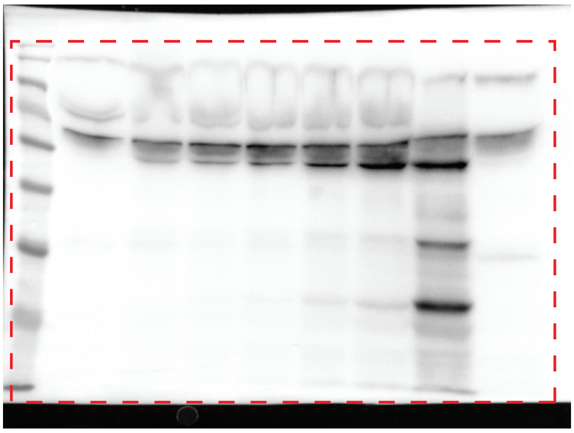

Figure 1B: Anti-Myc immunoblot

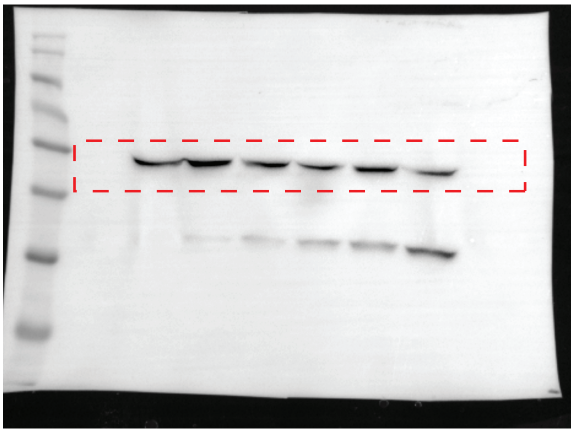

Figure 1B: Anti-Actin immunoblot (after Anti-Myc blotting)

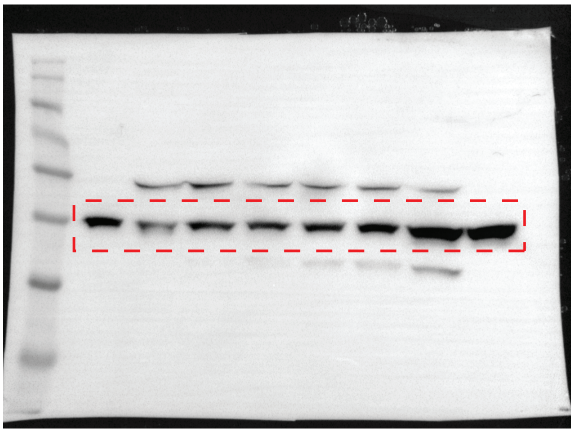

Supplement: Figure 1—source data 1. [file elife-64631-fig1-data1.pdf]
