# Supplementary material for: In vivo proteomic mapping through GFP-directed proximity-dependent biotin labelling in zebrafish
Source: eLife. 2021 Feb 16;10:e64631. doi: 10.7554/eLife.64631 (PMC7906605; doi:10.7554/eLife.64631)

Figure 2 - source file 1

Figure 2C: Streptavidin-HRP blot

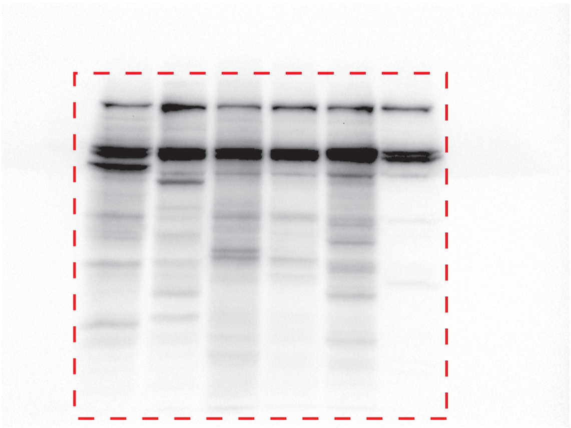

Figure 2C: Anti-Actin immunoblot

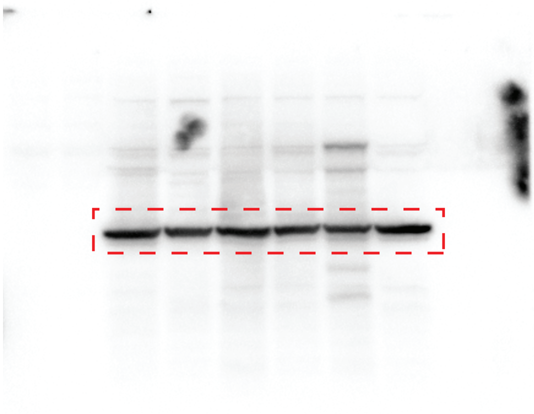

Figure 2D: Streptavidin-HRP blot

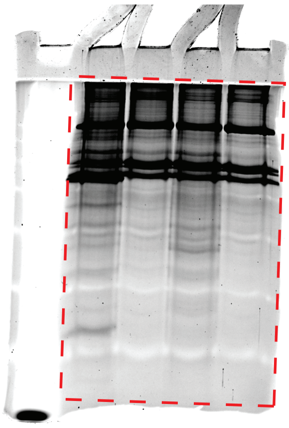

Supplement: Figure 2—source data 1. [file elife-64631-fig2-data1.pdf]
