# Supplementary material for: In vivo proteomic mapping through GFP-directed proximity-dependent biotin labelling in zebrafish
Source: eLife. 2021 Feb 16;10:e64631. doi: 10.7554/eLife.64631 (PMC7906605; doi:10.7554/eLife.64631)

Figure 4 - source file 1

Figure 4B: Streptavidin-HRP blot

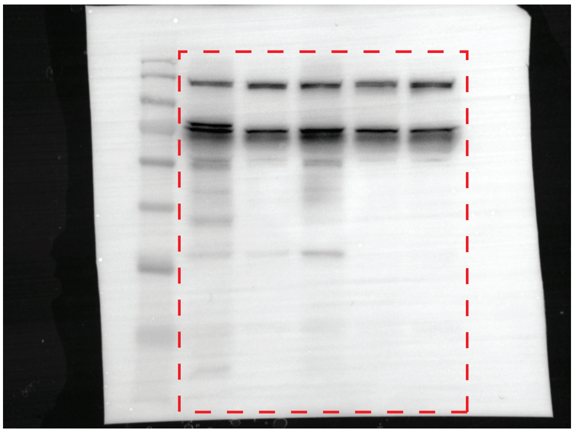

Figure 4B: Anti-GFP and Anti-Myc immunoblot

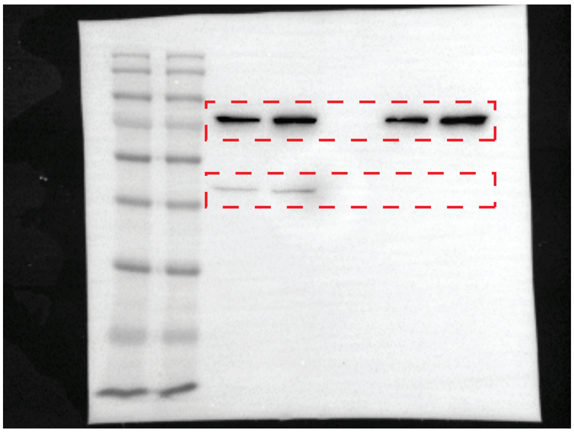

Figure 4B: Anti-Actin immunoblot

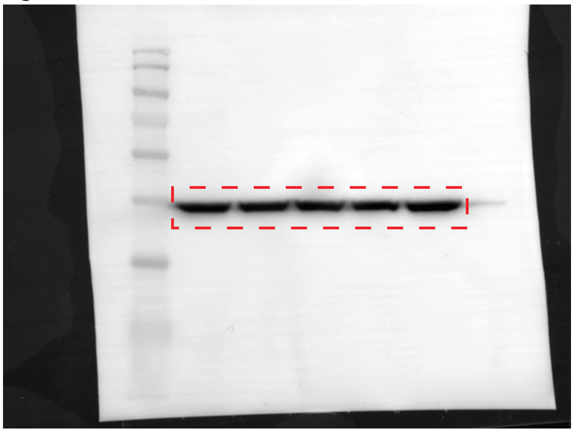

Figure 4 - source file 1

Figure 4C: Streptavidin-HRP blot

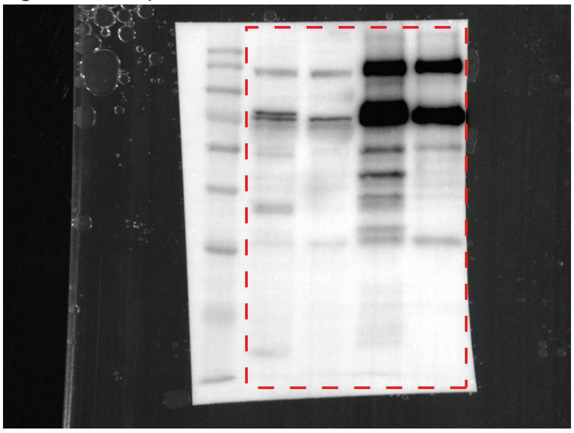

Figure 4C: Anti-Actin immunoblot

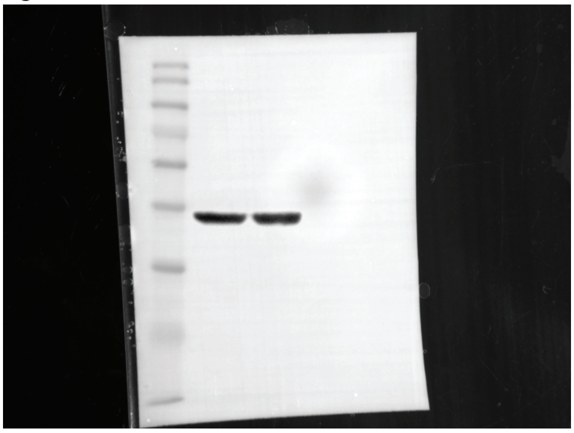

Figure 4C: Anti-Cavin4b immunoblot

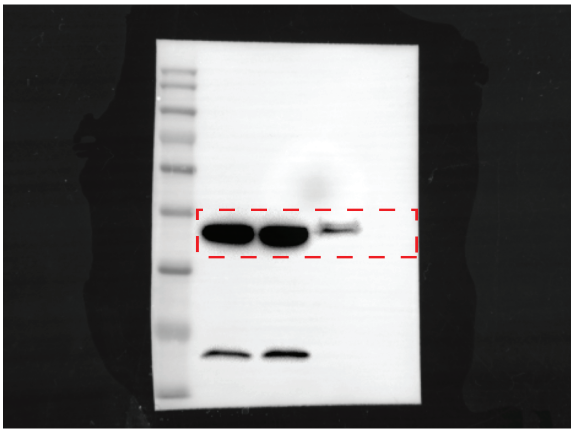

Figure 4C: Anti-GFP immunoblot (after anti-Cavin4b blotting)

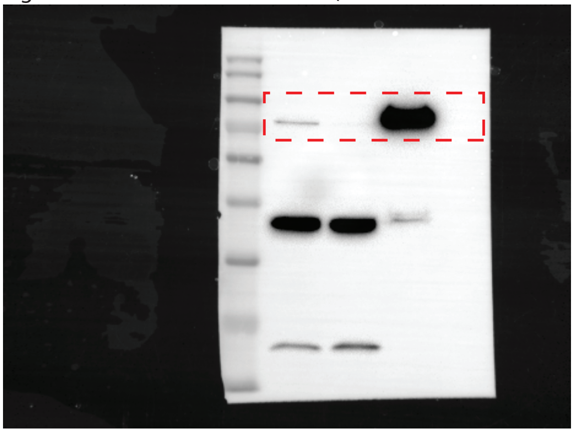

Figure 4C: Anti-Myc immunoblot, short exposure (after anti-Cavin4b and anti-GFP blotting)

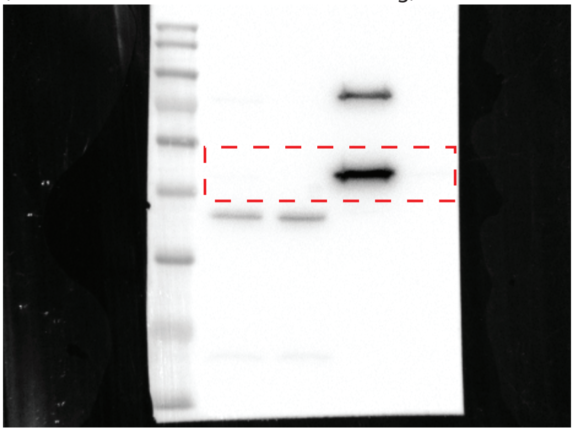

Figure 4C: Anti-Myc immunoblot, long exposure (after anti-Cavin4b and anti-GFP blotting)

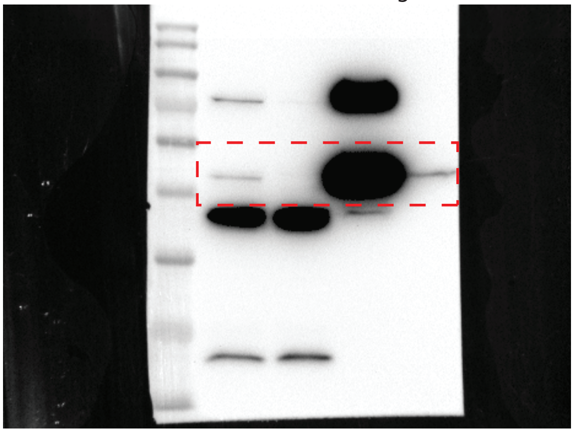

Supplement: Figure 4—source data 1. [file elife-64631-fig4-data1.pdf]
